# Trends in added sugar supply and consumption in Australia: there is an Australian Paradox

**DOI:** 10.1186/1471-2458-13-898

**Published:** 2013-09-30

**Authors:** Alan W Barclay, Jennie C Brand-Miller

**Affiliations:** 1 Australian Diabetes Council, 26 Arundel Street, Glebe 2037, NSW, Australia; 2 School of Molecular Bioscience, Biochemistry Building, G08, The University of Sydney, Sydney 2006, NSW, Australia

**Keywords:** Public health, Sugar, Obesity, Food supply

## Abstract

In 2011, Barclay and Brand-Miller reported the observation that trends in refined sugar consumption in Australia were the inverse of trends in overweight and obesity (*The Australian Paradox*). Rikkers et al. claim that the *Australian Paradox* is based on incomplete data because the sources utilised did not incorporate estimates for imported processed foods. This assertion is incorrect. Indeed, national nutrition surveys, sugar consumption data from the United Nations Food and Agricultural Organisation, the Australian Bureau of Statistics and Australian beverage industry data all incorporated data on imported products.

## The Australian Paradox has not been refuted

In the July 2013 issue of *BMC Public Health*, Rikkers et al. [1] attempt to estimate Australian refined sucrose supply and consumption over recent decades. They conclude that it is not possible to produce a reliable and robust estimate because of 'data limitations and a lack of current data sources’. Nonetheless, their analysis suggests that *imported foods* are now a greater contributor to intake of refined sucrose than they were in the past. Common sense would suggest that’s true because over the past decade we have imported more foods in general, but this finding does not prove that added sugars intake from all sources is now higher than in the past. Indeed, new data indicate that Australia now *exports* more foods and ingredients containing refined sucrose than 10 years ago [2]. There is evidence that not only Australians, but Americans are consuming less refined sugars than a decade ago [3].

In 2011, Barclay and Brand-Miller [4,5] reported three separate lines of evidence indicating downward trends in added sugars intake over the same timeframe that the prevalence of overweight and obesity among Australians had dramatically increased. We referred to this inverse relationship as the *Australian Paradox*. Rikkers et al. claim that the *Australian Paradox* is based on incomplete data because the sources utilised did not incorporate estimates for imported processed foods. This assertion is incorrect. Indeed, national nutrition surveys, sugar consumption data from the United Nations Food and Agricultural Organisation (FAOStat), the Australian Bureau of Statistics (ABS) and Australian beverage industry data *all* incorporated data on imported products. The apparent consumption data presented in the *Australian Paradox* covered 1980–2003, the most recent data at the time. Although the data for the 4-year period 1999–2003 now appear to have been underestimated by FAOStat, they do not alter the underlying trend – per capita sugar consumption is still lower than it was in 1980, and during that time frame obesity rates trebled.

Rikkers et al. have also misinterpreted the results of national nutrition surveys in 1983 and 1995 by confusing total sugars with added sugars. These surveys indicate the percentage of energy from *total* sugars (a measure that includes the naturally-occurring sugars in fruit, vegetables and dairy foods) remained either the same or decreased from 1983 to 1995 (depending on the age group) [6,7]. However, the surveys demonstrated declines in “sugary products” that contribute refined added sugar against increasing intakes of fruit and vegetables, implying that the absolute intake of refined added sugars had declined over time.

Australian beverage industry data also suggest that the total amount of added sugar consumed in the form of soft drinks decreased from 1997–2006 [8]. Unfortunately, Rikkers et al. interpret the change in the volume of beverages as equivalent to change in sugar consumption, failing to recognise a decline in the *concentration* of added sugar in soft drinks. Manufacturers now sell soft drinks with as little as 3-5% sucrose vs 10-12% in the past. This critical information is not encapsulated by volume sales data, but by data on amounts of sugar used by the beverage industry (Figure six in the *Australian Paradox*[4]).

The failure to recognise declining sugar concentration in Australian beverages may also apply to estimates of the sucrose content of imported foods. Indeed, it is not possible to determine precisely what proportions of imported soft drinks, chewing gums, chocolate and confectionery are manufactured with non-nutritive sweeteners and low digestibility sweeteners (e.g., polyols). We do know that 'low sugar’ and 'no added sugar’ products have become increasingly popular [9] and these are more likely to be imported than manufactured in Australia. The figure of 30 g sucrose/day from imported foods is therefore likely to be an overestimate.

**Figure 1:**
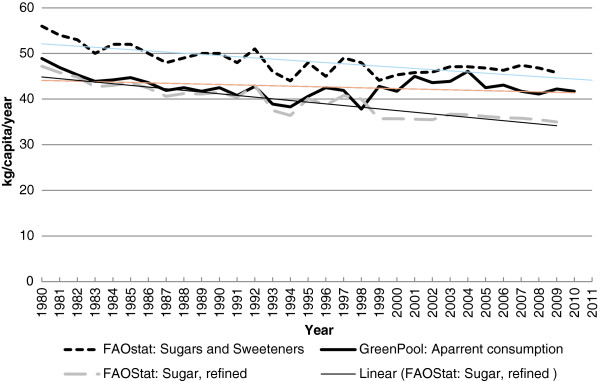
**Comparison of estimated per capita apparent consumption of sugar for Australia from 1980–2011 **[2,10]**.**

The analysis by Rikkers et al. makes much of imported sources of sugar, but overlooks the export of sugar as both a value-added ingredient, and in certain categories of food that are high in added sugars (e.g., dairy). Historically, apparent consumption data from ABS has included both imports and exports in processed foods. FAOStat data for Australia are almost identical to ABS data until 1998–99 when reporting ceased (Figure 1), implying similar methodologies. The most recent FAOStat data for Australia show that sugar availability has continued to decline (Figure 1). The Green Pool analysis [2] extended the ABS apparent consumption of sugar data series from 1999 to 2011 (Figure 1). Their detailed analysis included 173 categories of imported products and 120 categories of exported products, while Rikkers et al. included fewer than 20 food categories and overlooked exports of value added ingredients. The Green Pool analysis concluded that apparent consumption of sugar declined from 1980–2011, i.e., a conclusion that is similar to the most recent FAOStat data.

Other limitations should be noted. In their analysis, Rikkers et al. were obliged to make assumptions about the cost of imported food items in order to derive an estimate of amount consumed. However, imported goods vary markedly in price depending on country of origin, but can be much more expensive than the local product (up to 10-fold more per litre in the case of soft drink). Underestimating the cost of imported products will overestimate the amounts consumed and therefore overestimate the sugar content from imported products. Similarly, food wastage is now higher than in the past with 34% of food now wasted at the consumer level [11]. National nutrition survey data, as cited in the *Australian Paradox*, provide the most precise data on food actually consumed.

Finally, Rikkers et al. should heed their own conclusion that we should not make too much of incomplete data. Thankfully, reliable data on the intake of sugars by Australians will be generated by the 2011–12 National Nutrition Survey due for release in 2014 [12].

## Competing interests

Alan W Barclay is the Head of Research at the Australian Diabetes Council (ADC) and Chief Scientific Officer at the Glycemic Index Foundation (GIF). He has been with the ADC since 1998 and the GIF since 2001, and is a founding director of the latter. Directors are unpaid. Both companies are not-for-profit and their primary objectives are to help people prevent and manage diabetes. Via these companies Alan works with food industry to lower the glycemic index and load of foods and beverages. Companies are charged a fee for re-formulation advice. Advice is specific to carbohydrate containing foods and as such the foods are a mixture of sugars and starches. Alan is a co-author of a book on the prevention and management of diabetes that focuses on decreasing dietary glycemic load as a primary method of improving glycaemia. Alan is a co-author of The Australian Paradox paper referred to in the Rikkers et al. study.

Jennie Brand-Miller is President and Director of the Glycemic Index Foundation and a glycemic index testing service at the University of Sydney. She is co-author of *The Low GI Diet* (Hachette Australia) and other books that about the glycemic index of foods.

## Authors’ contributions

AWB and JCB-M co-wrote the original draft and edited the document. AWB prepared Figure 1. Both authors read and approved the final manuscript.

## Pre-publication history

The pre-publication history for this paper can be accessed here:

http://www.biomedcentral.com/1471-2458/13/898/prepub
